# Cache: Utilizing ultra-large library screening in Rosetta to identify novel binders of the WD-repeat domain of Leucine-Rich Repeat Kinase 2

**DOI:** 10.1186/s13321-025-01084-3

**Published:** 2025-09-25

**Authors:** Fabian Liessmann, Paul Eisenhuth, Alexander Fürll, Oanh Vu, Rocco Moretti, Jens Meiler

**Affiliations:** 1 Institute for Drug Discovery, Leipzig University, 04103 Leipzig, Germany; 2 Center for Scalable Data Analytics and Artificial Intelligence (ScaDS.AI) Dresden/Leipzig, Leipzig University, 04105 Leipzig, Germany; 3 Center for Structural Biology, Vanderbilt University, Nashville, TN 37235 USA; 4 Department of Chemistry, Department of Pharmacology and Institute of Chemical Biology, Vanderbilt University, Nashville, TN 37235 USA; 5 School of Embedded Composite Artificial Intelligence SECAI, 04105 Leipzig, Germany; 6 Faculty of Mathematics and Informatics, Faculty of Chemistry, Leipzig University, 04103 Leipzig, Germany

**Keywords:** Drug discovery, Ultra-large library screening, REvoLd, Rosetta, LRRK2, CACHE challenge

## Abstract

**Abstract:**

In this study, we present a pipeline for identifying novel ligands targeting the Tryptophan-Aspartate-Repeat domain 40 (WDR40) of Leucine-Rich Repeat Kinase 2 (LRRK2), a protein associated with Parkinson’s disease, as part of the first Critical Assessment of Computational Hit-finding Experiments (CACHE) challenge, a blind benchmark experiment for drug discovery. Mutations in this protein are the most common genetic cause of familial Parkinson’s disease, yet this target remains understudied. We conducted an ultra-large library screening (ULLS) of the Enamine REAL space using a newly developed evolutionary algorithm, RosettaEvolutionaryLigand (REvoLd), which allows for efficient screening of combinatorial compound libraries. The protocol involved refining the target structure with molecular dynamic simulations, identifying a binding site via blind-docking, and optimizing compounds through REvoLd, culminating in a manual selection amongst the top-scoring REvoLd hits. A single binder molecule was identified that derived from the combination of two Enamine building blocks. In the second round, derivatives of the hit compound were used as input for REvoLd to further sample within the Enamine REAL space. Ultimately, a total of five molecules were identified, from which three show a measurable dissociation constant K$_D$ value better than 150 $\upmu$ μm, showcasing the effectiveness of this approach. However, it also highlighted shortcomings, such as the preference for nitrogen-rich rings in the RosettaLigand scoring function.

**Scientific contribution:**

We introduce the first real-world application for REvoLd, an evolutionary docking algorithm enabling efficient ultra-large library screening for flexible protein targets. Our approach identified novel binders for the WDR40 domain of LRRK2 within the CACHE challenge #1, representing the first prospective validation of REvoLd. Here, we present a preparation pipeline to allow exploration of a large protein pocket with unspecific binding areas, and unlike prior brute-force docking efforts, our method integrates receptor flexibility and combinatorial chemistry optimization.

**Graphical Abstract:**

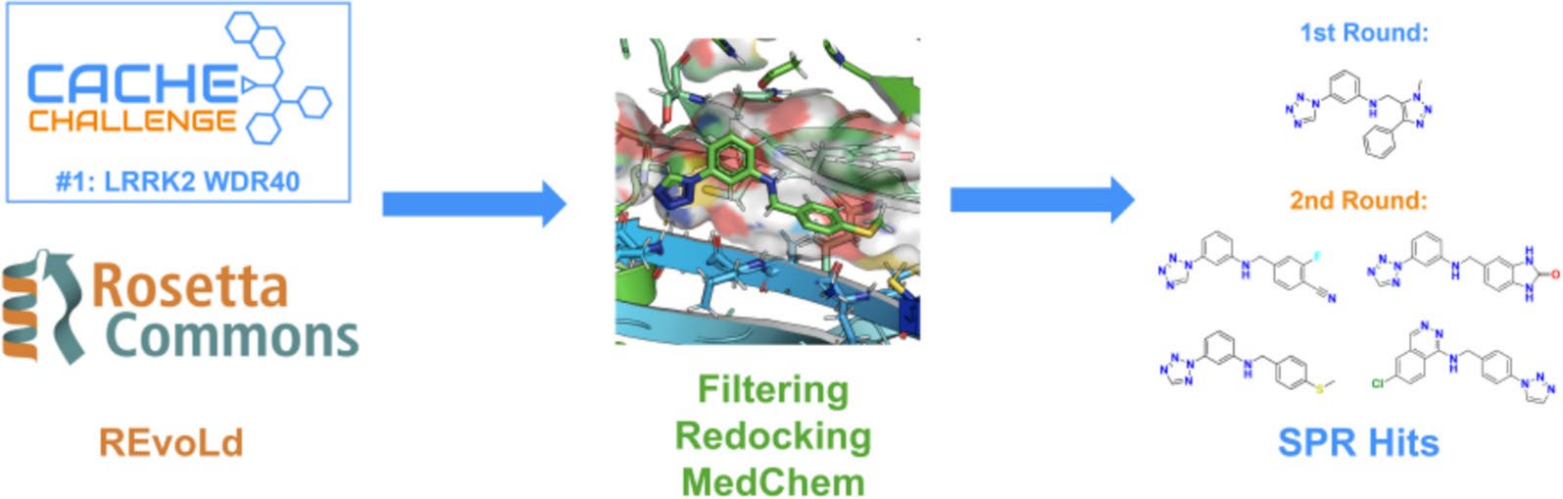

**Supplementary Information:**

The online version contains supplementary material available at 10.1186/s13321-025-01084-3.

## Introduction

Drug discovery, specifically the identification of hit compounds, has undergone a transformative evolution with the advent of readily available ultra-large compound libraries and associated computational screening and filtering algorithms [1, 2]. In recent years, there has been an explosion of computer-aided screening methods, driven by the integration of advanced computational techniques, artificial intelligence (AI), and machine learning (ML) [3–6].

This paradigm shift has enabled researchers to navigate the immense chemical space efficiently, probing libraries of compounds in the pursuit of novel therapeutic agents. Previously, a limitation had been the limited availability of chemical substances for acquisition compared to the size of the drug-like chemical space, which encompasses as many as 10^63^ potential compounds [7]. However, the development of make-on-demand libraries, such as Enamine’s REAL space and Otava’s CHEMriya, has mitigated this limitation by providing up to 64.9 billion readily accessible compounds [8–10]. Tailored computational methods (see below) can search this space to identify initial hit compounds with the activity of interest. This integration of computational methodologies with experimental validation represents a significant advancement in drug discovery, offering the potential to shorten development timelines and enhance success rates in identifying novel hit candidates.

However, the adoption of exhaustive enumeration methods, exemplified by approaches such as Lyu et al. in 2019 and colleagues [3, 11–13], remains challenging when dealing with libraries of such magnitude. These methods face significant computational hurdles in their application to billion- or trillion-sized ultra-large libraries (ULLs), which become computationally prohibitive, even when limited to one rigid protein model. RosettaEvolutionaryLigand (REvoLd) was developed to address the time and computational constraints associated with exhaustive screening of large compound libraries, particularly when including consideration of receptor flexibility [14]. Unlike brute-force methods that enumerate all molecules, REvoLd uses an evolutionary algorithm to explore chemical space by leveraging the combinatorial nature of these ULLs. The algorithm starts with molecules randomly constructed from the substrates and chemical reactions of the library. These molecules are then docked with ligand and receptor flexibility by RosettaLigand [15, 16]. During the following evolutionary optimization, substrates and reactions are exchanged and modified to iteratively optimize the resulting docking scores, yielding an enriched list of hit candidates [14].

In the realm of computer-aided drug discovery, benchmarking exercises similar to the Critical Assessment of protein Structure Prediction (CASP) [17] have taken place, but prospective experiments are scarce (such as Drug Design Data - D3R [18, 19]). Addressing this gap, the Critical Assessment of Computational Hit-finding Experiments (CACHE) challenge was introduced, envisioning a dynamic platform for comparing and enhancing small-molecule hit-finding algorithms. Drawing inspiration from the success of CASP in advancing protein structure prediction, the CACHE challenge stands as a unique undertaking, seeking not only to refine computational methods, but also to integrate rigorous experimental testing of the predicted ligands [20]. In the initial challenge, the aim was to predict hits for the Tryptophan-Aspartate-Repeat domain 40 (WDR40) of the Leucine-Rich Repeat Kinase 2 (LRRK2), recognizing its pivotal role as a compelling target in the context of Parkinson’s disease (PD). Mutations in LRRK2 associated with PD are known to promote the formation of LRRK2 filaments, which enhance pathological interactions with microtubules [21, 22]. Importantly, WDR40 domains, identified as both disease-associated and druggable, present an untapped avenue for therapeutic intervention [23]. Although substantial research has focused on inhibitors for the kinase domain of LRRK2, with several compounds in preclinical stages, most kinase inhibitors failed to inhibit the oligomerization of the protein on microtubules and stabilize the closed conformation [24, 25]. Positioned juxtaposed to the kinase domain, the WDR40 domain represents a promising unexplored frontier for novel therapeutic strategies in the treatment of PD. Recent structural insights highlight the importance of compounds stabilizing the open form of LRRK2 in inhibiting the pathogenic formation of LRRK2 filaments (PDB ID: 7LHT [26]). Additionally, the experimental determination of the WDR40 domain structure in 2020 has set the stage for further exploration, and hit discovery (PDB ID: 6DLO [27]).

This paper presents the pipeline methodology, results, and analysis of REvoLd as applied during the CACHE challenge #1, highlighting the computational strategies used for ULLS in Rosetta. The focus lies on the identification and characterization of a novel binder with promising affinities for the WDR40 domain of LRRK2. Through a combination of REvoLd and standard ligand docking in Rosetta, we provide insights into the binding mechanisms and potential implications for therapeutic development. Notably, the results highlight the discovery of an initial potential binder that underwent further chemical optimization, yielding additional four promising subsequent candidate molecules. This advancement holds significant promise for therapeutic development in targeting LRRK2 and is simultaneously the first experimental validation of REvoLd.

## Material and methods

### Compound database and library preparation

The CACHE challenge #1 was split into two rounds: After a hit identification round (round 1), successful teams were invited to a hit expansion phase (round 2). The combinatorial library specification for the Enamine REAL space was obtained directly from Enamine LTD under an academic non-disclosure agreement. This database consisted of lists of reactions (building rules) and associated reactants (building blocks/substrates). When fully enumerated these combinations add up to 19 548 368 812 compounds for the first round and to 30 789 836 702 for the second round. Reactions with two and three substrates were defined in SMARTS format (building rules) and substrates with their defined usage in the building rules were provided in SMILES format. All SMARTS and SMILES were combined into two tab-separated text files and provided as input for REvoLd [14].

RDKit (version 2022.09.1 [28]) was utilized to combine the substrates to complete molecules following the building rules. During all redocking steps, selected compounds and their respective 2D representations were prepared following the BCL (version 4.3.0) [29, 30] and RosettaLigand protocols (Rosetta version 2022.45 - 3.13 and REvoLd Rosetta version 2021.40 [15, 16, 31]).

### Ensemble of conformations for docking through molecular dynamics simulation

The crystal structure of the WDR40 domain of LRRK2 was obtained from the Protein Data Bank (PDB ID: 7LHT, [26]). The local conformational space around the experimental structure was explored with a molecular dynamics (MD) simulation. Proteins, optimized point charge (OPC) water, and ions were modeled with the FF19SB force field in AMBER [32]. Cl^-^ and K^+^ ions were added to reach a physiological salt concentration of 150 mM. The system was first minimized for 5000 steps using steepest descent followed by 15 000 steps of conjugate gradient minimization. During heating, the protein backbone and side chain atoms and water were restrained to their starting coordinates with harmonic force constants of 10 $\hbox {kcal mol}^{-1} {\text{\AA }}^{-2}$ and 5 $\hbox {kcal mol}^{-1} {\text{\AA }}^{-2}$, heated to 10 K over 10 000 steps with a step size of 0.1 fs using constant boundary conditions and Langevin dynamics with a rapid collision frequency of 10 000 p$^{-1}$s. The system was then heated to 100 K over 500 000 steps in 50 ps with constant volume dynamics and the collision frequency set to 1000 p$^{-1}$s and, finally, to 303 K over 1 000 000 steps with constant pressure dynamics and anisotropic pressure scaling turned on, while the positional restraints on the system were gradually removed. The system was then run with the protein held fixed for another nanosecond at 303 K. Production MD was conducted in three replicates. Each replicate was run for 1.5 $\upmu$s at 303 K using a step size of 4 fs with hydrogen mass repartitioning, constant pressure periodic boundary conditions (NPT system), semi-anisotropic pressure scaling, and Langevin dynamics. MD trajectories were analyzed using CPPTRAJ (version 18.0) and VMD (visual molecular dynamics; version 1.9).

The models obtained from the MD trajectories were clustered based on C$\alpha$-root-mean square deviation (RMSD) with DBSCAN [33] with sample minimum of 4 and $\epsilon$-value of 1.4 Å. For docking purposes, the resulting cluster centers were stripped of water and ions. All in all, eleven protein models were selected. Subsequently, a brief energy minimization was conducted in Rosetta to ensure the stability and suitability of the structures for further analysis within the Rosetta3 suite [34, 35]. The clustering information and the representative PDB files are provided in the supplementary.

### REvoLd as evolutionary docking algorithm to sample the chemical space

To obtain putative hit compounds, the recently developed evolutionary algorithm REvoLd was deployed [14]. It requires as input a receptor structure, the location of the binding site, and the definition of a combinatorial library (here, the Enamine REAL space). The standard protocol from the original publication [14] was used. In short, the protocol selects 200 random molecules (seed population) and docks them using RosettaLigand. The resulting initial set is reduced to 50 molecules using a rank-based selection. Through 30 iterative cycles, this population is increased to several hundred compounds through mutation (changing a substrate or reaction) and crossover (exchanging substrate between promising compounds). The parameters of these operations are chosen to ensure a balance between optimizing well-scoring molecules (local optimization), optimizing and maintaining medium scoring molecules (escaping local minima), and exploring chemical space to unveil new scaffolds. In each run, a rapid divergence from the seed population is observed: Within a few generations, a chemical diversity between following generations is observed, which is dependent on the seed population. REvoLd uses a normalized ligand binding score as its fitness metric. In this approach, the interface energy is normalized for molecule size by dividing the score with the square root of the number of non-hydrogen atoms, resulting in a measure called "ligand interface delta square root normalized", short lid_root2. At the end of each generation the population gets reduced through rank-based selection. This is repeated until 30 generations are complete and all docked molecules are reported (including discarded molecules). Several independent runs are performed instead of one long REvoLd run. Despite beginning from entirely distinct seed populations, the score trajectories converge on nearly identical paths: rising rapidly before plateauing, which demonstrates robust optimization independently of the initial population. REvoLd was used during both rounds with slightly different settings.

In a first exploratory docking, the grid size for ligand docking was increased from 10 Å to 12 Å and allowed movement distances from 0.1 to 0.5. Furthermore, instead of 150 sampled protein-ligand poses, 200 were tested to allow for more thorough testing. The exploratory docking included 44 runs (four per protein structure) and resulted in 75 600 compounds. A follow-up run with 18 runs per protein structure resulted in 358 713 sampled compounds.

In the second round, 304 pre-selected compounds were utilized as the starting population and 214 000 compounds sampled in 60 runs (20 for three protein structures). In the corresponding sections of Results and Discussion it will be explained in more detail.

### First filtering step

Molecules were filtered based on derived Lipinski’s Rules of Five [36] and a drug-likeness filtering protocol recommended by the CACHE committee, employing a traffic light (TL) scoring scheme where lower scores indicate higher desirability (see Table 1) [20, 37]. Chemical properties (molecular weight between 150 and 500 Da, logP between -1 and 5, number of rotatable bonds less than or equal to 10, and hydrogen bond donors/acceptors less than or equal to 5 and 12, respectively, for the second round, the number of hydrogen bond acceptors was limited to less than or equal to 10) were computed using RDKit (version 2022.09.1 [28]) and served as hard filters. Polar surface area (PSA) and and fraction of sp$^3$ carbon atoms (Fsp$^3$) were also calculated but not used as hard cut-off filters. Each compound’s TL score was summed, and those exceeding a total score of 2 were removed from further consideration. Additionally, molecules containing Pan Assay Interference Compounds (PAINS) substructures were excluded [38]. Here, the PAINS filter catalog from RDKit was utilized.

**Table 1:** Traffic light scoring scheme for the first CACHE challenge defined by the CACHE organizers

TL value	(K$_D$)	(Solubility)	LogP	MW	PSA	Rotatable bonds	Fsp^3^
0	(<20)	(>100 $\upmu$M)	<3	<400 Da	<120 Å^2^	0–7	>0.3
1	(<70)	(>50 $\upmu$M)	<4	<500 Da	<140 Å^2^	8–10	<0.3
2	(<100)	(<50 $\upmu$M)	>4	>500 Da	>140 Å^2^	$\ge$11	<0.2

Each compound is scored based on multiple physicochemical and structural properties, including dissociation constant (K$_D$ ), solubility, logP (lipophilicity), molecular weight (MW), polar surface area (PSA), number of rotatable bonds, and the fraction of sp^3^ carbon atoms (Fsp^3^). A traffic light score (0, 1, or 2) is assigned, where lower scores indicate more desirable properties for drug discovery, and higher scores highlight less favorable characteristics. For filtering, K$_D$ and solubility were excluded (marked with parentheses)

### Second filtering step through redocking with RosettaLigand

The best scoring REvoLd compounds underwent redocking using RosettaLigand (Rosetta version 3.13) [15, 16, 31], where 50 conformers per ligand were generated using BCL (version 4.3.0) [29, 30]. The binding pose from REvoLd was utilized as the starting docking pose for redocking in the established RosettaLigand protocol. For every compound a total of 600 docking poses were generated. In the first round, 10 000 compounds were selected for redocking and 7000 compounds in the second round. The interface score between the docked compound and protein was calculated alongside the lid_root2 term for normalization. A RMSD-score plot was then generated based on the best scored docking pose, which was considered in the filtering process. To limit the calculation time, only poses with an interface score better than -10 REU were considered for calculating the ligand RMSD.

### Automated and manual compound selection

Redocked molecules were filtered based on docking scores. Here, a lid_root2 cut-off of better than −3.5 was set, which removed 30% of the molecules (equaling 3128 compounds in the first round and 2238 in the second round). Additionally, the difference in docking score between REvoLd and the redocking as well as between the best ten redocked poses was calculated. A difference in the docking score in both calculations with more than 0.8 lid_root2 was a filter criterion to exclude a compound. This threshold was chosen to prioritize compounds with stable binding predictions while filtering out potential docking artifacts. Large variations in docking scores may indicate unstable binding modes, sensitivity to minor conformational changes, or inconsistencies in scoring functions rather than strong, well-defined interactions. In the first round, out of 10 000 redocked compounds 4,015 passed the filtering criteria, while in the second round, out of 7000 redocked compounds only 453 passed. All selected compounds were clustered into 100 groups by k-means based on Tanimoto similarity calculated with extended connectivity fingerprints with a diameter of 4 (ECFP4) with RDKit [39, 40]. Lastly, the most promising compounds were manually selected. Therefore, the docking pose of the best ten docking models were visualized in PyMOL (The PyMOL Molecular Graphics System, Version 2.4.1 Schrödinger, LLC.). This step was supported by the calculated RMSD-score plot with respect to the best docking pose. Several criteria for selection of promising compounds were included: Convergence to a single binding mode (presence of an energy funnel), quality of ligand-protein interactions (fitting of the ligand at the binding site, orientation of hydrophobic moieties, protruding of functional groups, amount of hydrogen bonds and unsaturated hydrogen bond donors), and drug-likeness of the molecule (avoidance of undesired structural features like long alkyl chains, terminal alcohol moieties, polyphenols, chelating agents, dicarboxylic acids, ketones). Consistency in the top scoring docking models is likely to be a predictor of a true ligand binding pose [41]. Finally, the chemical structure was analyzed for undesired moieties/properties in hits, leads, and drugs, like too many nitrogens, bi-aryl structures, silicon-containing compounds, covalent warheads and azides [42, 43] (see Supplement List). As requested by the CACHE committee, final sets of molecules were submitted for potential acquisition. In the first round, 150 compounds were submitted for potential acquisition, while in the second round 72 compounds were selected.

### Expanding the hit space

The experimentally verified hit compound from the first round of experimental screening (see Results and Discussion) resulted from a two-substrate reaction. To expand the candidate list for submission in the second round, a combinatorial expansion was employed by replacing each of the two substrates with every replacement option in the Enamine component list for this reaction whilst keeping the other one in place. This resulted in 18 193 compounds which were docked with RosettaLigand and filtered by a lid_root2 score threshold of −3.5, yielding 304 molecules. These molecules were then used as the starting population for REvoLd instead of the random population. For every run, 200 of these 304 were picked randomly and added 50 random molecules from the whole Enamine REAL space. A total of 60 REvoLd runs were conducted with such pre-seeded populations, resulting in 214 602 molecules before filtering.

### Off-target and screening

As defined by the CACHE committee after the first round, binding to the Proline-Tryptophan-Tryptophan-Proline 1 (PWWP1) domain of the Nuclear Receptor Binding SET Domain protein 2 (NSD2) should be avoided. Therefore, to adhere to this recommendation, all 757 compounds identified in the second round were docked against this off-target. The experimentally determined structure of NSD2 (PDB ID: 7MDN, [44]) was prepared following the standard ligand docking protocol in Rosetta. In short, the structure was stripped of water and bound ligands and one domain of the tetramer energetically minimized in Rosetta. The compounds were docked with the same parameters as described for the WDR40 domain of LRRK2 and the starting point defined as the binding pocket of the experimentally determined ligand. Off target compounds were identified by lid_root2. Cutoff values for lid_root2 depend on the target and the respective score distribution (see Supplement Fig. 9). For PWWP1, a cutoff of −3.5 struck a balance between excluding compounds that had predicted off-target binding, while preserving diversity in the accepted set. 628 compounds passed the off-target filter, equaling 83% of the selection. Subsequently, compounds passing the off-target filtering were then ranked and filtered as well as selected as in the first round.

### Make-on-demand synthesis and experimental validation

Selected compounds were ordered and synthesized by Enamine LTD. using their in-house pipelines [9]. 101 out of 109 and 37 out of 45 compounds ordered were successfully synthesized. Around 1 mg of each compound was dissolved in DMSO. Both identity and purity were verified by liquid chromatography-mass spectrometry as reported by Enamine LTD. Compounds were tested by the Structural Genomics Consortium, University of Toronto as described in [37]. Several methods and orthologue testing strategies were used to identify and validate potential hit compounds including surface plasmon resonance (SPR), differential scanning fluorimetry (DSF), Fluorescence Polarization (FP) Assay, Dynamic Light Scattering (DLS) and $^{19}$F-NMR spectroscopy. For experimental details see Li et al. [37].

## Results and discussion

**Fig. 1:**
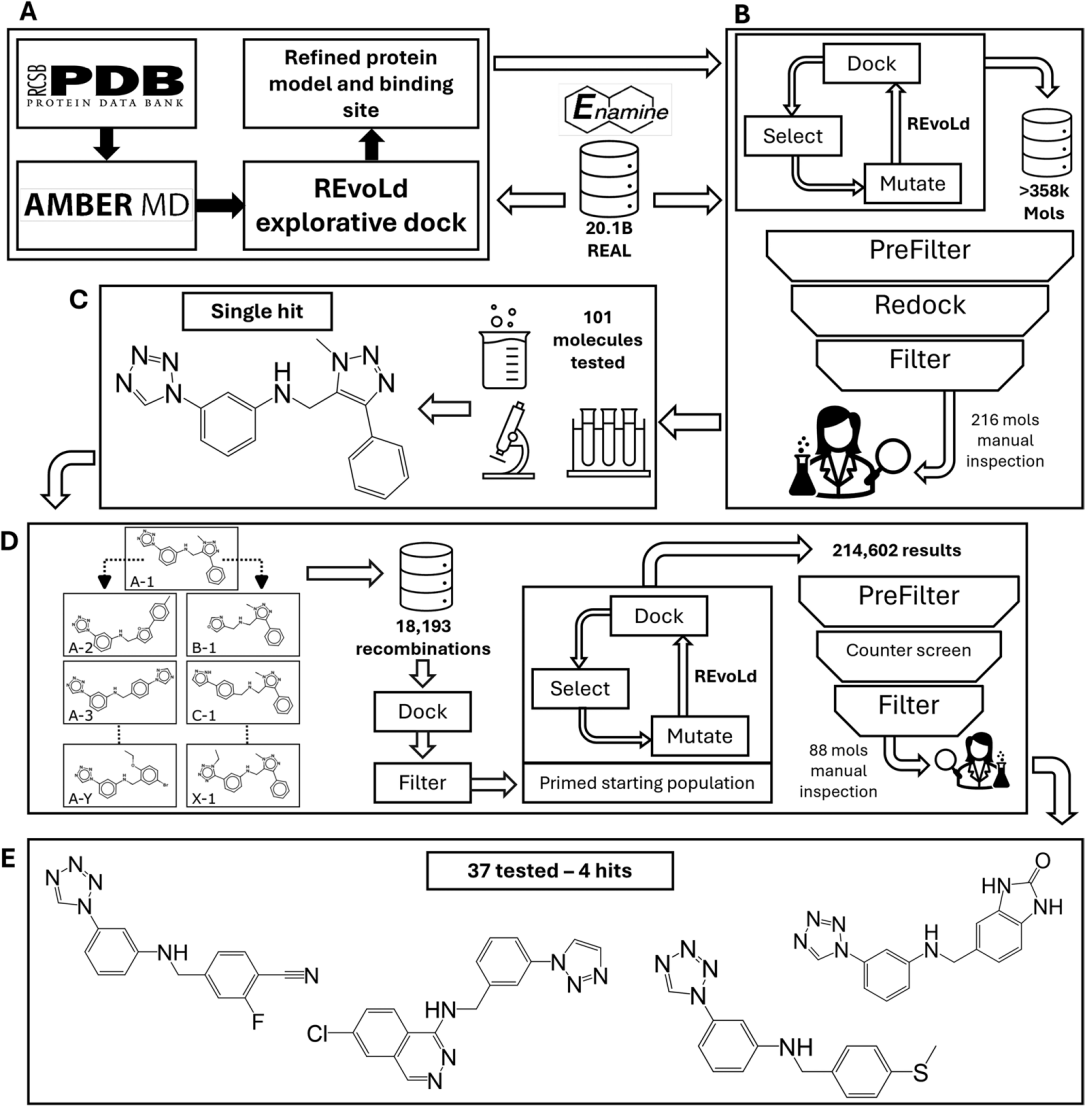
Workflow for CACHE challenge #1. **A** MD simulation generated an ensemble of target structures, and consensus docking identifies likely binding sites within the central cavity. **B** In the first round REvoLd was employed for ultra-large library screening and implemented a prefilter of the results with respective compound scores and physicochemical properties based on Lipinski’s Rule of 5. The top scored compounds were redocked and filtered for score and PAINS substructures. Manual inspection included clustering with k-means similarity, a manual hit selection with visual inspection of docked poses. **C** After submitting 101 compounds, the experimental validation and screening resulted in a single hit. **D** In the second round we started with the brute force recombination of all building blocks and redocking. Based on a score-filter, the best results were used as the starting population for REvoLd which resulted again in 214 602 enriched mols. Again, a filtering pipeline was used to prepare a manual hit picking party. **E** The testing of 37 compounds in the final round resulted in 4 additional preliminary hits

Our comprehensive multi-step workflow for discovering novel inhibitors targeting the WDR40 domain is shown in Fig. 1.

### Using molecular dynamics simulation to sample the conformational space of WDR40

**Fig. 2:**
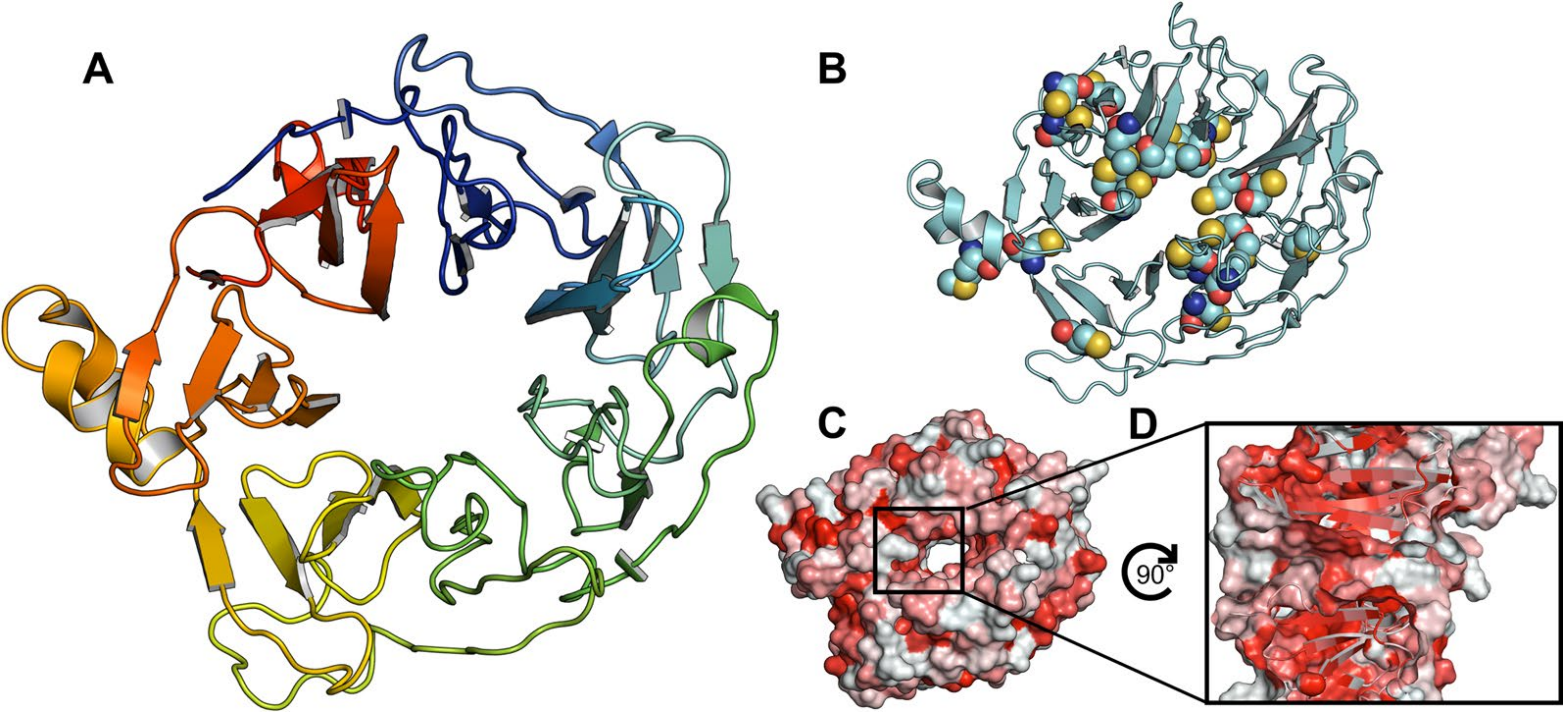
Structure of the LRRK2 WDR40 domain. **A** Overview of seven-bladed β-propeller fold. **B** Cysteines and methionines (spheres) are concentrated around the central cavity. **C** Hydrophobic residues (red) line the central cavity. **D** Cross section of central pocket, showing hydrophobic patches

The virtual screening campaign started by examining the two available experimentally determined structures for LRRK2 WDR40. Although the full LRRK2 structure (PDB-ID: 7LHT), possessed a worse resolution (3.5 Å) than that of the isolated WDR40 domain (PDB-ID: 6DLO; 2.7 Å), the WDR40 domain from the full LRRK2 structure was selected as the primary model due to its more complete loop regions. Despite the disparity in resolution, the C$\alpha$-RMSD of approximately 0.8 Å between the LRRK2 full structure and the isolated WDR40 domain underscores their structural similarity and interchangeability. LRRK2 WDR40 is a 361 amino acid long seven-bladed β-propeller domain [45] (see Fig. 2A). The central cavity has a diameter of approximately 12 Å and a depth of 26 Å. Recently, several related WDR40 structures with bound ligands were published, showing binding sites throughout the entire central cavity [46–48]. A structural analysis of the LRRK2 WDR40 domain identified a potential binding site, that is populated with sulfur-containing residues, forming a distinct arrangement of polar and non-polar patches (see Fig. 2B–D). For this reason, the central cavity was chosen as the focus of our ligand binding studies.

To explore the conformational flexibility within the binding site and increase the probability of investigating relevant pocket geometries, MD simulations were conducted. These simulations provided an ensemble of starting points with varying loop orientations. After clustering the protein-only structures, eleven cluster centers were selected as representative models. By increasing structural diversity while focusing on the most representative models, the efficiency of the screening process is expected to improve, along with the likelihood of identifying potent inhibitors by targeting diverse yet specific conformations of the binding site. The observed variations, especially in the connecting loops, underscore the importance of considering different loop conformations in the drug design process. An average C$\alpha$-RMSD of around 1.5 Å across structures indicates moderate structural fluctuations that primarily affect the flexible loop regions while maintaining the overall integrity of the β-propeller core. This level of variability suggests that the binding site undergoes conformational changes that may impact ligand binding. As the later identified binding site for the ligands are in the outer region of the β-propeller and interacting with some loop regions, this highlights the relevance of considering these dynamics and flexible areas.

### Exploring the binding site with extended REvoLd docking

**Fig. 3:**
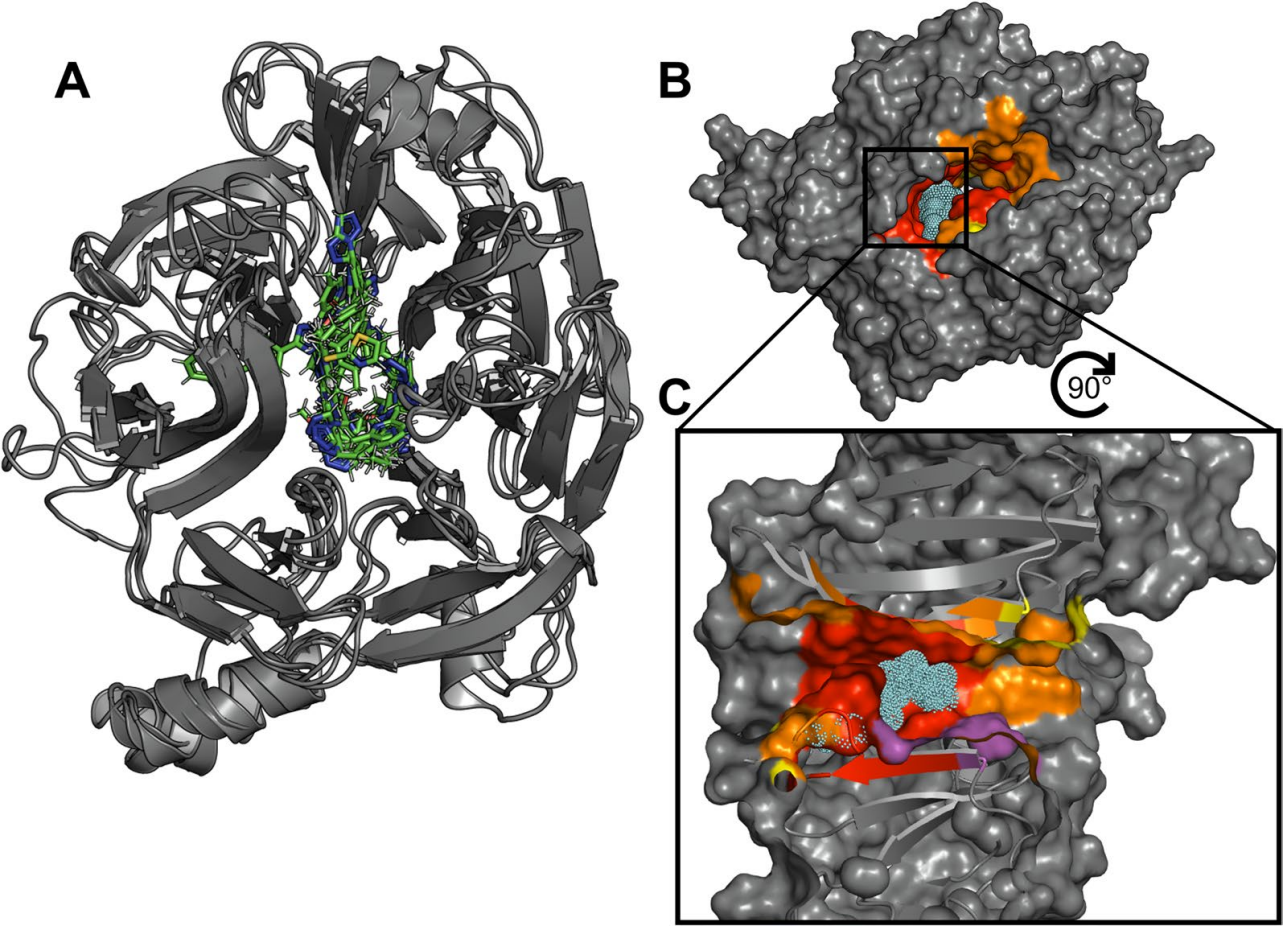
Investigation of the binding site. **A** Best ligands from the extended docking overlapped in a potential binding site. **B** Binding site residues colored by the number of pre-screening ligands found in interaction - red: more than 500 ligands interacting, purple: 400-500, orange: 400-200, yellow: 200-100, cyan dots: representative ligand **C** Cut through this area

To conduct a thorough exploration of the binding site within the central cavity, an extensive sampling with REvoLd was conducted. Initial ligand placement was at the geometric center of the cavity, but the allowed binding site definition (grid and box size for docking) was expanded to include the full cavity. Furthermore, the number of generated protein-ligand complexes during docking was increased from 150 to 200 to accommodate the large volume [15]. From a series of 44 runs (four per protein model from the MD simulation), yielding approximately 75 500 total compounds, the 1000 top scoring compounds were extracted and analyzed. To identify areas within the central cavity to focus on, the number of docked compounds within 4 Å of each residue was counted. This analysis identified two distinct regions with a high frequency of ligand binding interactions (see Fig. 3). The area defined by these patches was used for the following screenings as starting points to allow the decrease of sampling during docking and therefore, decrease computation times.

### Virtual screening with REvoLd for identifying promising compounds and subsequent filtering

Several ULLS runs with REvoLd were initiated using the clustered eleven models derived from the MD simulations, and the two starting positions previously identified. These runs used the default settings as described in the original publication and summarized in the methods section [14]. They differ from the initial explorative runs by a reduced number of protein-ligand complexes generated during docking (150 as suggested by DeLuca et al. [15] instead of 200), smaller grid and box sizes for docking to only consider ligands at the binding site, and a higher number of REvoLd runs.

For each protein structure 18 runs were conducted, all in all 198 additional REvoLd runs, resulting in 358 713 molecules. Together with the 44 explorative ones, over 434 000 compounds were optimized in total. More than 91% of all investigated compounds passed first filtering, as described in the methods section. The top-performing 10 000 compounds, based on lid_root2, underwent redocking in RosettaLigand. To optimize computational efficiency, a detailed analysis of the top-performing 10 000 compounds and their respective protein conformations was conducted to identify the most relevant structural models for redocking. A single structural model accounted for 65% of the top performing compounds, prompting its utilization for subsequent redocking procedures. This protein structure originated from the largest cluster from MD. Although no significant differences in cavity size and hotspot residue exposure were observed, local differences in loop orientation change the volume of smaller pockets. For redocking the sampling protocol was expanded to 600 docking poses for a more comprehensive evaluation. Furthermore, as a countermeasure against artifacts, compounds were filtered by consistency in redocking, by examining the difference in docking scores within the best ten docking poses and to the original REvoLd docking score. A difference of more than 0.8 for the lid_root2 score was considered indicative of a docking artifact, and those ligands were discarded.

**Fig. 4:**
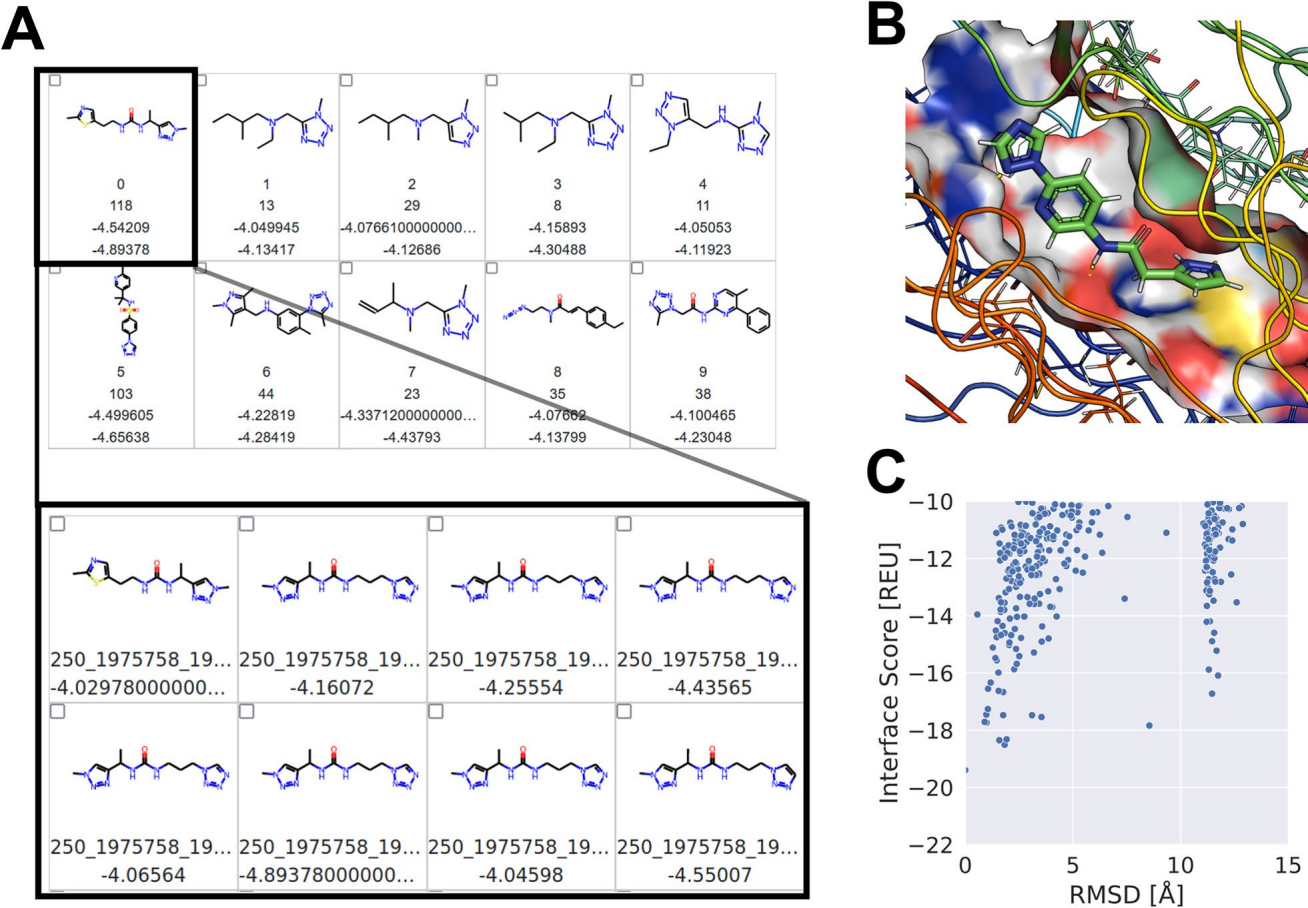
Selection pipeline and exemplary results. **A** The different clusters were visualized with the respective cluster center in a Jupyter Notebook. Below the chemical structure, the cluster number is shown as well as how many compounds are included in this cluster. To highlight the results, the lid_root2 score of the representative as well as the best scored compound within the cluster are shown. By selecting one or several clusters, the individual compounds in the selection are visualized in another window. Here, the single compounds with internal naming and their lid_root2 score are shown. **B** For promising compounds from the selection in **A** the top ten docking poses were visualized in PyMOL and evaluated. This step incorporated medicinal chemistry expertise for evaluating binding energy distributions. **C** For each investigated compound, the RMSD-score plot was calculated. Here, the ligand RMSD to the best scored pose was calculated. A funnel indicated preferable docking results, and several funnels suggest multiple plausible binding modes

Subsequently, all 4015 compounds meeting the criteria were clustered based on Tanimoto similarity. Cluster numbers ranging from 50 to 200 were explored, with lower cluster numbers grouping highly diverse compounds together, while numbers above 125 resulted in several clusters containing only single compounds. Ultimately, 100 distinct clusters were chosen and visualized. A subsequent expert-driven hit-picking session with medicinal chemists led to the selection of 216 final compounds. Here, to generate a maximally diverse compound set, 1-3 compounds per cluster were selected. Within each cluster, compounds were chosen based on a comprehensive evaluation that included the docking score, chemical properties, traffic light score, and structural considerations - an illustrative example of this process is depicted in Fig. 4. To ensure the robustness of the final selection, the docking results of these compounds were visualized and analyzed in PyMOL. Here, the best ten docking poses by lid_root2 score were analyzed and their binding evaluated. Key criteria for evaluation included the generation of hydrogen bonds, proper orientation towards and within the previously identified binding patches, fitting within sub pockets, and the consistency of the binding mode across all ten complexes. Compounds that consistently fulfilled these criteria were considered promising candidates.

### Compound clustering, selection, and procurement process

From the pool of selected compounds, a subset of 150 was earmarked for potential acquisition and cost quotations. Here, the Tanimoto similarity between the selected 216 compounds was calculated and compound pairs with high similarity selected. Starting with the pair with the highest similarity, the compound with the better score was selected. This exclusion process was repeated until only 150 compounds were remaining (see Table S1). This final list was submitted for cost quotation and the synthetic accessibility was verified for 143 molecules. To stay within budget, the four most expensive compounds (those exceeding $150 including import fees) were excluded. The exclusion process of the most similar compound pairs as described before was repeated until the compound set was within budget. An order of 109 compounds was placed, of which 101 were successfully synthesized, resulting in a synthesis success rate of 92.7% (See Table S2). These compounds were then experimentally tested by the CACHE organizers [37].

### Exploration of the identified binder scaffold with REvoLd

**Fig. 5:**
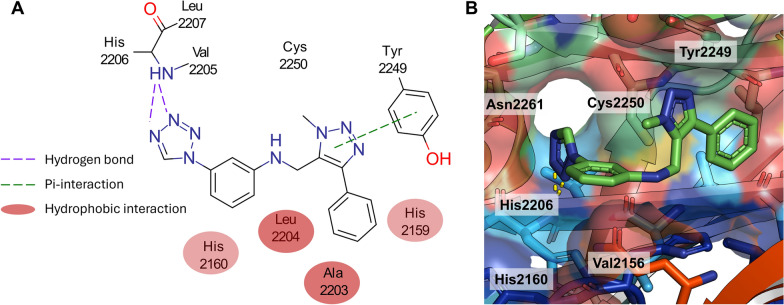
Predicted binding mode of compound **A**. **A** The interaction diagram highlights many different interactions between the ligand and the binding site, especially to the backbone of Val2205. Protein residues are represented by circles, while protein-ligand interactions are depicted as lines. The colors of both residues and interactions vary according to the interaction type, as indicated in the legend. **B** The three dimensional representation of the best docking pose of compound **A** (green color) shows the complex binding towards the main interaction partners (cartoon representation in rainbow coloring, protein-ligand hydrogen bonds are shown as dotted lines)

The experimental validation with SPR resulted in one weak binder **A**, *N-[(1-methyl-4-phenyl-1 H-1,2,3-triazol-5-yl)methyl]-3-(1 H-1,2,3,4-tetrazol-1-yl)-aniline*, with a K$_D$ value of 59 $\upmu$ m (Fig. 5). Compound **A** has a weight of 332.4 Da, one H-bond donor and eight H-bond acceptors, as well as five rotatable bonds and a *logP* of 2.1. In the following paragraph, the proposed binding mode of the initial hit will be explained, which guided the selection of the next generation of compounds.

The model indicates two key interactions for the binding of the weak binder **A**. The first is a $\pi$-$\pi$-interaction between the triazole ring and phenyl ring of Tyr2249. The second and probably most important one, is a hydrogen bond formed between the tetrazole and the backbone amide hydrogen of His2206. This special kind of hydrogen bond is a so-called bifurcated or three-centered hydrogen bond While their energy is only between 50 and 60% of a canonical hydrogen bond (one acceptor and one donor group), they play an important role in protein interactions [49]. In this case it consists of two hydrogen bond acceptors, nitrogen 2 and 3 of the tetrazole ring and the NH group of the amino acid. The angles between N-H-N are 149° and 167°, which are in an acceptable range for these kinds of interactions [50, 51]. These two significant interactions are accompanied by hydrophobic interactions with the two phenyl motifs of compound **A**. The terminal phenyl ring interacts with the hydrophobic regions of Ala2203 and His2159. The aniline ring shows interaction with Leu2204 and His2160. Furthermore, the model provides an indication of the only weak binding affinity of our compound **A**. There is an excess of six possible H-bond acceptors and, most importantly, one H-bond donor, the NH group of the aniline motif. Those potentially unsatisfied hydrogen-bonding interactions can negatively affect ligand binding affinity, although the presence of water molecules may partially compensate for them. However, despite the limitation in H-bonding, this compound has a good predicted binding score and geometric fit in the binding pocket (see Fig. 5B).

Based on the identified hit, a scaffold exploration was carried out. The hit molecule was generated from a two-component reaction specification. Each of the two substrates forming the initial hit were recombined with each possible partner for the building reaction to generate 18 193 compounds. These molecules were docked with RosettaLigand against the three structural models which resulted in the most compounds generated with REvoLd after filtering. A consensus score of the top scoring models was generated and the standard deviation calculated. The previously described filtering pipeline took place, with an additional focus on the different scores in the various models. Compounds with a standard deviation of more than ±0.5 in lid_root2 score were excluded. 304 compounds remained with an enrichment of tetrazole containing compounds.

To further enhance the candidate pool, the selected compounds were utilized as seed population for REvoLd, forming the first generation. An additional 214 000 compounds were generated in the Enamine make-on-demand space, iteratively optimizing the discovered motifs. After initial filtering, the top 7000 compounds by score were chosen to balance computational feasibility and maintain chemical diversity for redocking in the three structural models. Applying the same filtering criteria ultimately reduced this set to 453 compounds.

For the second round of this CACHE challenge, non-specific and off-target activity was monitored by binding to the PWWP1 domain of NSD2. The first hit showed a small amount of off-target binding affinity by SPR. Consequently, a counter screen to identify compounds with potential interactions was implemented. Compounds were redocked with RosettaLigand into an experimentally determined structure of PWWP1 with a bound ligand (PDB ID: 7MDN), and compounds scoring lower than $-$3.5 lid_root2 were excluded (see Supplement Fig. 9). Of the 757 combined compounds from partner enumeration and REvoLd expansion, 628 passed the off-target counter screen and were subsequently clustered based on Tanimoto similarity. As described before, a hit-picking session supported by medicinal chemists rigorously examined the remaining compounds, leading to the identification of 88 promising candidates. Finally, the top-performing 72 compounds were submitted for cost quotations, with an order placed for 45 compounds (See Table S3). As the goal of the second round is to find similar molecules, compound selection was performed on the basis of lid_root2 scoring to WDR40, rather than pairwise similarity.

### Experimental results and in-silico binding site investigation

**Fig. 6:**
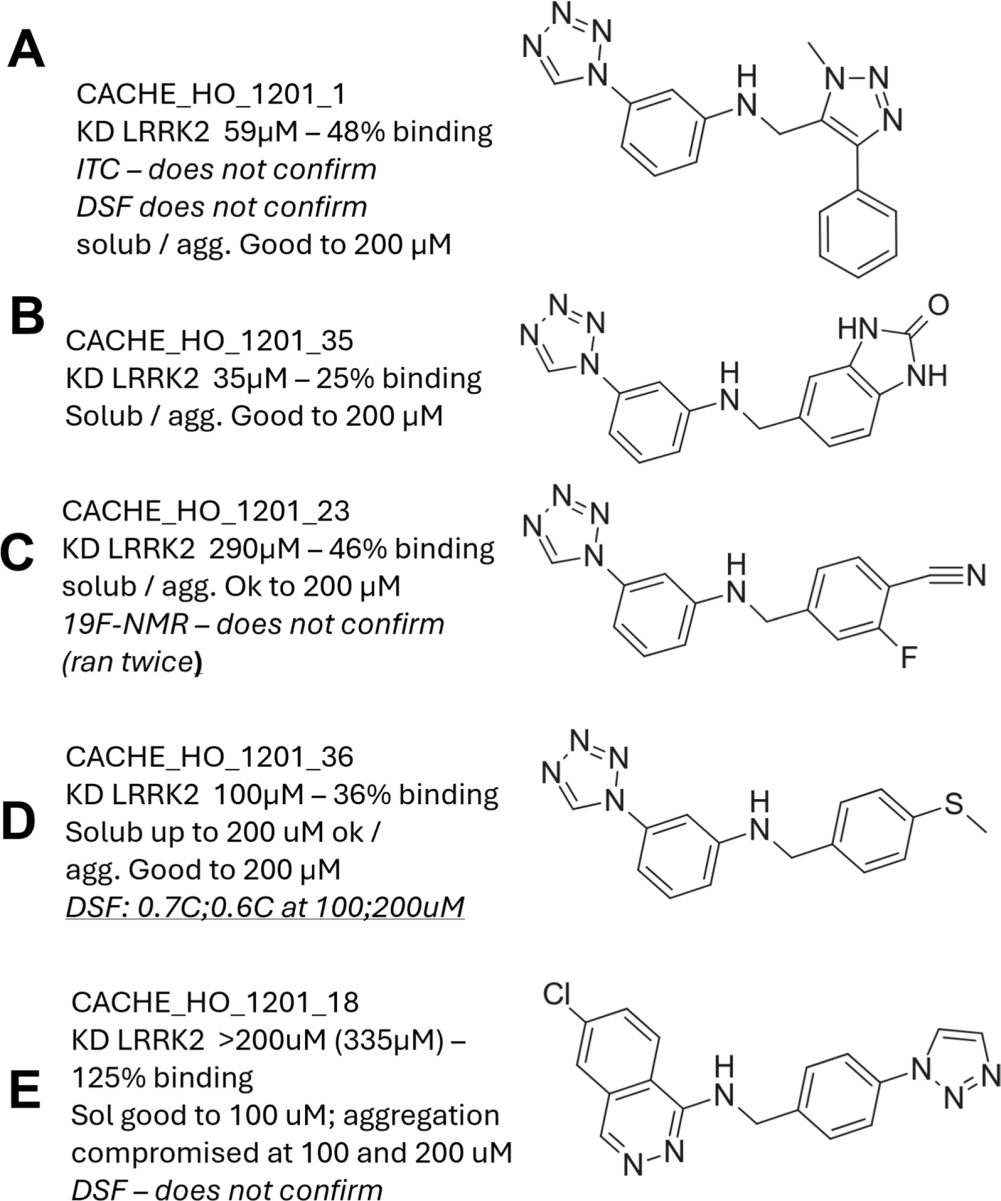
Experimental results and compound structures. The experimental results of the second round for the identified hits were sent as an overview page by the CACHE organizers (see also Figure S4). For each compound **A-E** their respective K_D_ value, percentage of binding and orthogonal assay results as well as solubility results are reported as well as their chemical structure

The experimental evaluation of the compounds in the hit-expansion round resulted in four novel potential binders (see Fig. 6 and see Supplement Fig. 12). Compared to our first round hit **A** (K$_D$ = 59 $\upmu$ m), two of them showed a significantly lower affinity with K$_D$ values of 290 $\upmu$ m (**C**) and 335 $\upmu$ m (**E**, with tendencies to unspecific binding and aggregation) and one hit **B** showed an increased binding with K$_D$ = 35 $\upmu$ m. The fourth compound **D** ranked in between with a K$_D$ of 100 $\upmu$ m, but was the only one that showed weak stabilization effects ($\Delta$ T$_m$ = 0.7 $^{\circ }\text {C}$) in differential scanning fluorimetry. None of the other compounds, including our first round hit, could be confirmed by orthogonal assays, including DSF, ITC or, in the case of **C**, $^{19}$F-NMR. For this reason, we chose compound **D** as an example to retrospectively investigate its predicted binding site and compare it with our first round hit **A**.The two compounds whose measured K$_D$ values exceed the 150 $\upmu$ m threshold (compounds C and E) should be regarded not as confirmed binders but rather as sub-optimal hits or false positives. Although they displayed weak interaction in the SPR assays, their affinities fall outside the CACHE‑defined “active” range.

**Fig. 7:**
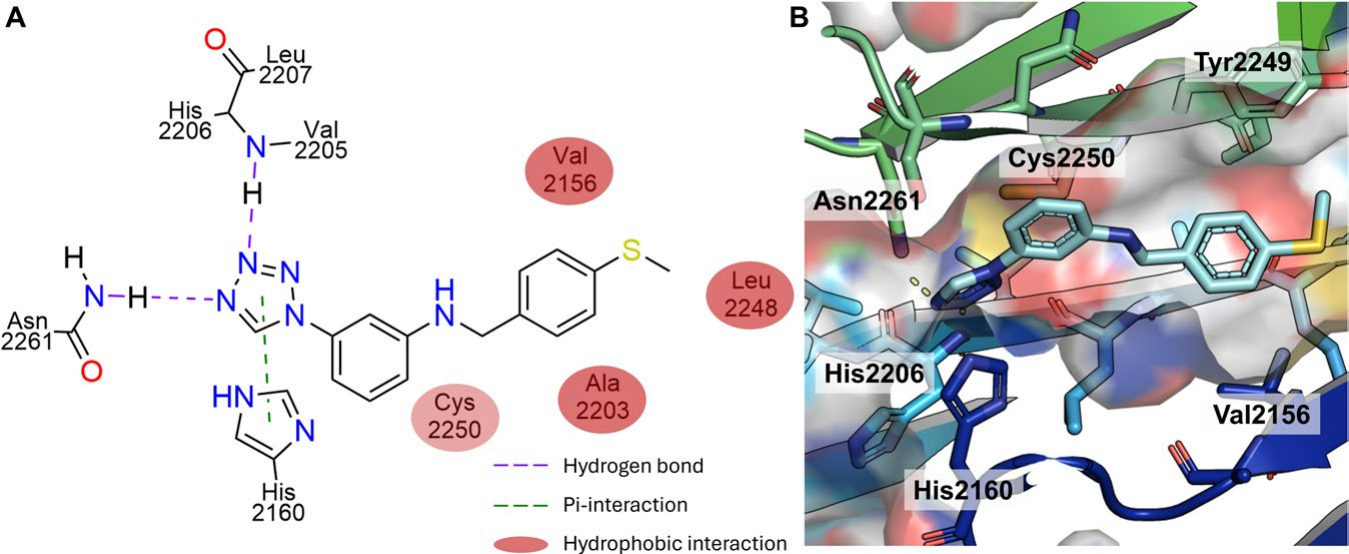
Predicted binding mode of compound D. **A** Protein-ligand interaction diagram for compound **D** shows a similar binding to the backbone of Val2205 and additional hydrogen bonds to Asn2261. Protein residues are represented by circles, while protein-ligand interactions are depicted as lines. The colors of both residues and interactions vary according to the interaction type, as indicated in the legend. **B** The three dimensional representation of the best docking pose of compound **D** (cyan color) towards the main interaction partners is shown in cartoon representation and rainbow coloring. Protein-ligand hydrogen bonds are shown as dotted lines

Compound **D**, *N-[4-(methylsulfanyl)phenyl]methyl-3-(1 H-1,2,3,4-tetrazol-1-yl)aniline*, has a weight of 297.4 Da, one H-bond donor and five H-bond acceptors, as well as five rotatable bonds and a *logP* of 3.0. It showed similarity to the initial hit **A** from the first screening (see Fig. 5). Three important interactions were identified, comprising two hydrogen bonds and one $\pi$-$\pi$-interaction. The first hydrogen bond is formed between nitrogen 3 of the tetrazole ring and the amino function of His2206. The second hydrogen bond is observed between nitrogen 4 of the same tetrazole and the amino function of the Asn2261 side chain. A potential $\pi$-$\pi$-interaction was identified between the tetrazole ring and the imidazole side chain of His2160. With these three interactions the tetrazole motif formed a distinctive interaction pattern that aligns the position of the ligand within the binding site and most likely has the greatest impact on its binding affinity and stabilization effect. The two phenyl rings and the methyl group of the thioether formed non-polar interactions. The aniline group interacted with the hydrophobic region of Cys2250, whereas the aromatic thioether interacted with Ala2203 and Val2156. The methyl group demonstrated interactions with Leu2248 (see Fig. 7).

In comparison, the initial hit **A** and compound **D** show similar interactions. There are three amino acids involved in both binding modes. Most importantly, His2206, which in both cases formed hydrogen bonds, His2160 and Ala2203. For both the tetrazole rings as H-bond acceptor was responsible for the formation of hydrogen bonds, while for compound **A** only His2206 was involved as H-bond donor for a bifurcated hydrogen bond, while compound **D** formed an additional canonical hydrogen bond with Asn2261. In addition, each ligand formed one $\pi$-$\pi$-interaction. The principal distinction between these two compounds is that compound **D** established all three directed interactions with its tetrazole and three different amino acids, whereas compound **A** interacted with the tetrazole to form the hydrogen bond and the triazole to form a $\pi$-$\pi$-interaction. This interaction pattern may explain the observed stabilizing effect of compound **D** compared to compound **A**.

**Fig. 8:**
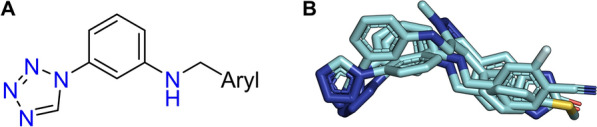
Pharmacophore model of hits found in SPR. **A** Based on the chemical structure, a simple pharmacophore of compounds **A-D** can be derived. **B** The overlay of compounds **A-D** extracted from their respective best binding pose underlines this model. A similar orientation of the tetrazole ring and the aniline motif is observed

The remaining three compounds (**B**, **C**, **E**) exhibited analogous interactions in their docked models, comparable to those observed in the two previously described ligands (**A**, **D**), including the unsatisfied hydrogen bonds. The four compounds with the highest binding affinity (**A** - **D**) share a common pharmacophore-like moiety, namely the terminal tetrazole ring linked to the aniline motif (see Fig. 8). Two of the four compounds (**B**, **D**) are from the initial scaffold exploration. The other two compounds are from the REvoLd run in the second phase (**E**, **C**). Interestingly, the weakest binder (**E**) lacks both the tetrazole ring and the aniline ring.

### Final evaluation and challenges identified in the screening pipeline

Our pipeline produced five preliminary hits out of 138 tested molecules. Three out of these five molecules had a measurable dissociation constant K$_D$ value better than 150 $\upmu$ m, meeting the threshold of the CACHE organizers [37], corresponding to a 2.17% hit rate. This falls below the reported average hit rates for ULLS of between 6.7% and 7.6% as reported in Sousa et al. [52], it remains notably higher than typical *in-vitro* high-throughput hit rates of 0.01–0.14% [53]. In the same publication, ULLS hit rates between 1% and 40% are reported, but these statistics vary considerably with the target and experimental validation protocols. To place our results in the context of the CACHE challenge, we compared our success rate with that of other teams. According to the CACHE organizers [37], 73 compounds in the first round achieved a K$_D$ values below 150 $\upmu$ m (as measured by SPR assays) across 23 groups, spanning teams with zero hits to teams with 11. The overall median was two hits per group, while this approach identified one. In the second round, 31 analogs from 18 remaining participants achieved a K$_D$ value below 150 $\upmu$ m, of which we submitted two. The median of hits per group is two compounds, again, while the best group identified five analogs. When looking at the hit rate of hits with K$_D$ value below 150 $\upmu$ m in respect of submitted compounds in round 2, we achieved 5.3% while the best group had 12.5%.

All approaches and results were reviewed, scored and ranked by an independent Hit Evaluation Committee. This approach and results positioned us in the mid-tier, achieving a 10th place ranking out of 23 participants. We successfully advanced to the second round with one compound, a milestone not reached by several other teams. In total we were able to identify five potential binders by SPR, one of which (**D**) also showed low stabilization in the DSF assay. However, this result is questionable and was not considered as orthogonal confirmation. The other ligands could not be confirmed in an additional orthogonal assay. However, the lack of precise data on binding kinetics and modes may limit precise statements on the respective performance.

Among the seven workflows that ultimately delivered the hit series in CACHE challenge #1, a surprisingly wide array of strategies was deployed. 19 of the 23 participants relied on physics‑based docking as their core screening engine, but beyond that point the methods diverged sharply. Twelve groups incorporated at least one machine‑learning component, and eight of those used deep‑learning–based docking [37]. Fragment‑centric approaches, either seeding the pocket with crystallographic or PDB‑derived fragments or transferring subpockets via structure‑based matching, were adopted in five workflows (notably FRASE‑bot and the POEM subpocket algorithm [54, 55]). Seven teams, including ours, simulated protein flexibility with MD to generate receptor ensembles for docking. Of these, three performed among the top-ranked teams. Manual medicinal‑chemistry curation was surprisingly rare. In fact, the overall challenge winner achieved top performance using only GNINA docking scores with minimal filtering or human intervention [56]. In this landscape, our approach stands out in two respects. First, it is the only pipeline to employ Rosetta–based preparation of the protein and docking within the Rosetta Suite. Second, whereas many teams used machine learning or deep‑learning models, a combination of de-novo generative methods followed by chemical similarity search, or fragment‑based approaches, our approach was unique in pairing an MD‑derived receptor ensemble with a true evolutionary search (REvoLd) of the full Enamine REAL space, without any preliminary ML filtering or fragment seeding [37]. This orthogonal, evolution‑driven approach yielded novel chemotypes and complementary insights into pocket dynamics, underscoring the value of diverse methodologies in ultra‑large‑scale virtual screening. More information for all participants, including the winning teams, can be found in [37, 54–58].

Overall, achieving 14 out of a possible 20 points in the CACHE challenge as the first practical application of the REvoLd algorithm establishes a solid foundation for future enhancements. In this rigorous competition, strict deadlines required swift decision-making and expedited *in-silico* screening and analysis, presenting both challenges and valuable lessons. While the accelerated timelines constrained our ability to conduct in-depth structure-activity relationship (SAR) exploration and utilize more comprehensive computational techniques, such as MD simulations for top ligands, these limitations illuminated critical areas for improvement. We were successful in establishing a screening pipeline with the novel algorithm REvoLd during the two rounds and prepared all the necessary scripts and evaluation tools throughout the process. This challenge facilitated the development of a robust pipeline for rapid compound characterization, improving the efficiency of computational predictions and validation processes under stringent time constraints. A key challenge encountered was the need to systematically integrate diverse compound selection criteria. For each investigated compound, several layers of information including various scores, individual score differences, physicochemical properties and more, could be gathered but for the final selection compromises must be made. For example, initially, it was planned to use MD simulations on the best ligands to investigate the binding in more detail. However, while inclusion of this information may help further improve the final results, the observed level of performance indicated it may not be critical for a successful screening campaign in time- and computation-limited conditions.

A key area for improvement is the RosettaLigand scoring function. Specifically, a bias toward certain compounds and chemical structures, in particular tetrazoles and multiple nitrogens, was noticed (see Supplement Figs. 10, 11). Of 94 submitted compounds with a tetrazole motif in the first round, 73 were from our group. Scoring functions may disproportionately favor certain interactions, leading to an overestimation of compound binding affinities. This becomes especially problematic when screening or optimizing within billion large spaces [10]. To mitigate these issues, several strategies can be considered. One is combining several scoring functions into a consensus method [59]. This integration of multiple computational predictions can potentially balance the biases of any single scoring system. Additionally, scoring methods based on first principles like free-energy perturbations can mitigate unintended bias as well [60]. Another strategy involves not strictly selecting the top-scoring compounds but instead choosing a set of high- to mid-high-scoring candidates to explore a broader range of chemical space. This was proposed by Lyu et al. [10] in their analysis of screening artifacts. Due to the scoring bias for nitrogens, the top-scoring compounds contained mostly tetrazoles. However, as one proceeds down the list the fraction of tetrazole containing compounds drops: Out of the best 10 000 investigated compounds, 6117 contained a tetrazole motif. Going further, from the best 50 000 compounds more than half had the aforementioned moiety, while the following 50 000 only contained 20 000 tetrazoles (see Supplement Fig. 11). Instead of focusing only on the best 10 000 compounds, a broader score range combined with compound and moiety similarity filtering can mitigate the risk of only capturing artifacts. These adjustments aim to enhance the robustness and accuracy of our screening process in future challenges.

An additional drawback of the evolutionary optimization protocol is its potential interaction with binding mode optimization, which warrants further investigation. While REvoLd attempts to optimize binding affinity, it does so by using RosettaLigand docking score as a proxy. However, as RosettaLigand is a stochastic sampling algorithm, it is possible that REvoLd is not optimizing for actual binding affinity but only for molecules that simplify RosettaLigand docking. This is indicated by the high number of hydrogen bond donors and acceptors in result compounds. If a molecule is covered in potential interaction partners, a sampling-based docking algorithm can more easily identify promising binding modes which form at least some hydrogen bonds. This would mean the evolutionary algorithm of REvoLd is actively exploiting the scoring function. To mitigate this overfitting, future iterations will (a) incorporate a refined scoring function to more effectively penalize non-hydrogen bonding polar atoms during the docking process, (b) use consensus rescoring with an orthogonal engine (e.g., GNINA [56]), and (c) introduce pose-consistency filters to ensure that high scores reflect robust binding modes rather than sampling artifacts. These refinements should better align the evolutionary search with true binding affinity and diversify the physicochemical profiles of the top candidates. REvoLd’s evolutionary search, while capable of traversing ultra‑large combinatorial spaces, sampled only 0.0018% of the available compounds in Round 1 and 0.0007% in Round 2. To gauge whether this limited coverage contributed to missing chemotypes later validated by other CACHE teams, we computed Tanimoto similarities between our entire pool of 572 000 sampled molecules and the seven distinct binder scaffolds [37]. Several of our sampled compounds reached chemical similarity with two of the seven winning scaffolds yet were not advanced. This can be traced back to not just a scoring but also to post-selection. Here, compounding effect arose from our manual curation process. In our manual curation process, identified binders would have been discarded by our medicinal chemistry filters (e.g., the winning scaffold ID 1202 would have been omitted due to its phenolic liability [37]). Together with the Rosetta scoring bias toward nitrogen‑rich chemotypes and the finite sampling, this manual‑selection step likely eliminated genuine binders.

Finally, we analyzed the difference of all MD structures in respect to each other and to the original structure (PDB ID: 7LHT [26]). While the β-propeller remains highly similar and the positioning is conserved across the replicas, the loop regions are flexible. Particularly, the loop region spanning Ala2157 to Ile2167, which contains the interacting residue His2160 with its imidazole side chain and potential $\pi$-$\pi$-interaction with the tetrazole ring, exhibited notable flexibility. Here, the loop moves in and out of the binding site across the different replicas, increasing or decreasing the size of the pocket. This dynamic reshaping of secondary cavities underpins the improved docking of certain chemotypes in the MD-derived ensemble. During the redocking in Round 1 the MD derived model that yielded the highest number of top-ranking compounds exhibited a loop orientation closely resembling the crystal structure. To assess whether the MD step was essential, we performed a control docking of the three binders against the unmodified experimental structure. This control docking run on the experimental structure alone recovered two of the three hits. These results suggest that, although the MD conformational ensemble is broadly similar to the experimental structure, the minor side chain and loop adjustments captured in the MD snapshot were critical for identifying at least one of the three binders presented here. Still, even without this additional step in generating a conformational ensemble, binders can be identified. Consequently, the MD-derived ensemble provided valuable insights into the flexibility for hit identification.

## Conclusion

The CACHE challenge is an effort by the Target2035 initiative, which aims to identify medical probes for the entire human proteome within open science principles [20, 61]. CACHE provides a rigorous and comprehensive benchmark for prospective computational hit-finding algorithms and pipelines, with the goal of facilitating discovery of novel binders for any protein. This study presents a comprehensive multi step workflow in Rosetta for the CACHE challenge #1, targeting the WDR40 domain of LRRK2.

Our pipeline combined MD simulations, evolutionary optimization, classical ligand docking and filtering, and expert-guided selection. The pipeline identified a single hit in the first round, highlighting areas for further optimization. Due to limited structural data on the protein-ligand complex, key decisions in this pipeline were based on inferred binding site properties and predictive modeling. Whilst these assumptions were made to the best of our knowledge, they are potential sources of errors. During the second hit expansion round, we successfully discovered four additional potential binders building out from the initial binder, from which two showed a K$_D$ value better than 150 $\upmu$ m. The quality of the binders remains uncertain with only one of the hits was partly confirmed by an orthogonal assay. Given this limited validation of the identified hits, further biophysical validation, ideally through structural validation in co-crystallization or related techniques, will be essential prior to advancing these compounds in a medicinal chemistry campaign.

A retrospective analysis further highlighted potential issues in the applied scoring function which overrated hydrogen bonds and therefore, induced a bias towards nitrogen rich rings. These findings highlight specific areas for improvement, including the incorporation of multiple scoring functions, consensus scoring methods, and physics-based approaches such as free-energy perturbations. Despite pipeline limitations, the stringent deadlines of the CACHE challenge were met while successfully developing a streamlined workflow centered on Rosetta and REvoLd.

In summary, the presented screening approach, integrating advanced computational methods, expert-guided selection, represents a systematic and collaborative strategy for identifying both initial binders and expanding from known binders. Three potential ligands for the WDR40 domain of LRRK2 could be identified, providing valuable insights in the pursuit of novel therapeutic interventions for Parkinson’s disease.

## Supplementary Information

Supplementary material 1. Files S: All clustered PDB structures and clustering information from the MDsimulation.

Supplementary material 2. Table S1: All initial 150 selected compounds and their SMILES code.

Supplementary material 3. Table S2: All initial 109 ordered compounds and their SMILES code.

Supplementary material 4. Table S3: All improved 72 selected compounds and their SMILES code.

Supplementary material 5. Table S4: All improved 45 ordered compounds and their SMILES code.

Supplementary material 6. Supplement Fig. 9–12 (referred to as Appendix B (B1-B4) and Appendix C (Supplement list).

## Data Availability

Data is provided within the manuscript or supplementary information files. Additional publically available information from CACHE challenge #1: https://cache-challenge.org/results-cache-challenge-1.

## Code availability

Rosetta and REvoLd are available through the Rosetta repository https://github.com/RosettaCommons/rosetta.
