# Supplementary material for: Cache: Utilizing ultra-large library screening in Rosetta to identify novel binders of the WD-repeat domain of Leucine-Rich Repeat Kinase 2
Source: J Cheminform. 2025 Sep 25;17:145. doi: 10.1186/s13321-025-01084-3 (PMC12465524; doi:10.1186/s13321-025-01084-3)
Supplement: Supplementary file 6 — Supplementary material 6. Supplement Fig. 9–12 (referred to as Appendix B (B1-B4) and Appendix C (Supplement list). [file 13321_2025_1084_MOESM6_ESM.pdf]

Appendix B Supplement Figures

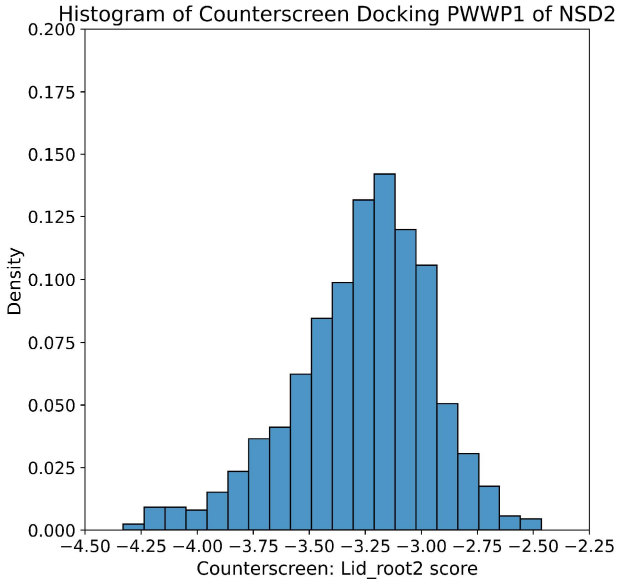

Fig. B1 Counter screen results for PWWP1 domain of NSD2. In the second round of the CACHE #1 challenge, off-target binding was assessed by RosettaLigand redocking of the most promising 757 compounds against the PWWP1 domain of NSD2. From the distribution of lid_root2 docking scores, it was determined that compounds with a score greater than -3.5 were unlikely to have above background binding to the off-target protein.

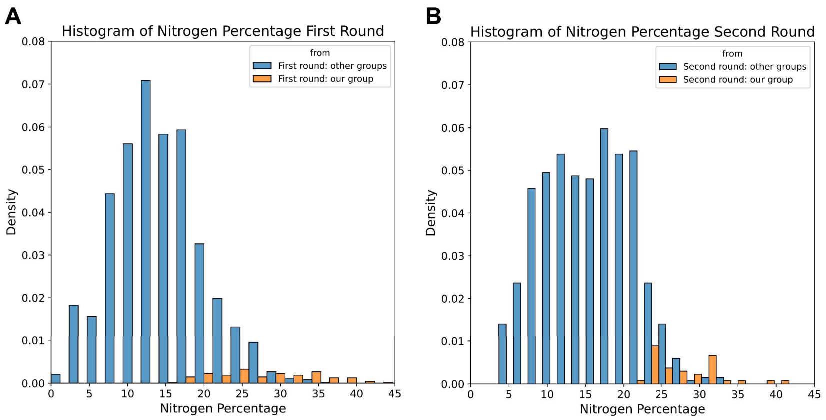

Fig. B2 Nitrogen proportion on heavy atoms for all compounds. A) In the first round our compounds are broadly distributed with respect to nitrogen proportion, though generally have a greater fraction of nitrogen than the compounds of other CACHE participants. B) During the second round, preservation of the tetrazole motif from the first round hit continues to result in higher-than average nitrogen fractions.

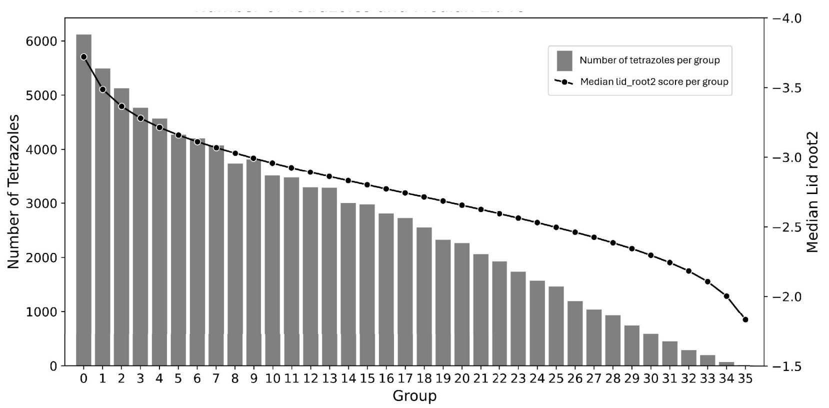

Fig. B3 Number of tetrazoles in 10 000 compound groups for the 358 713 compounds from the large virtual screening with REvoLd in the first round vs their median lid_root2. All 358 713 compounds were ranked by their lid_root2 score and grouped in 10 000 compound sets (group 0-35). The number of tetrazoles in this group is plotted as a gray bar plot (left x-axis) with their respective median lid_root2 score as black dot line (right-x-axis) in each respective compound group. A correlation between both is observable.

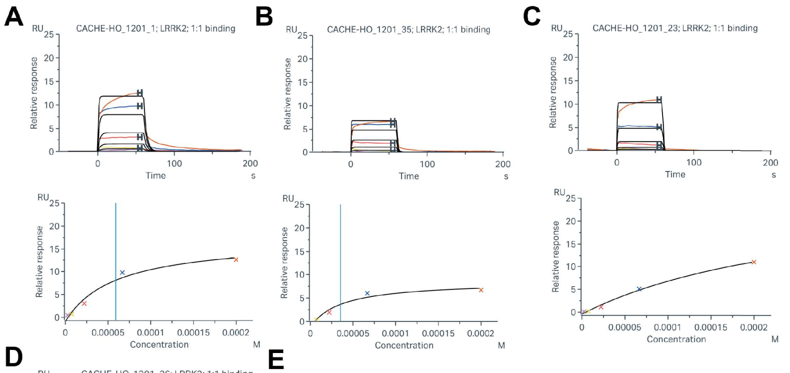

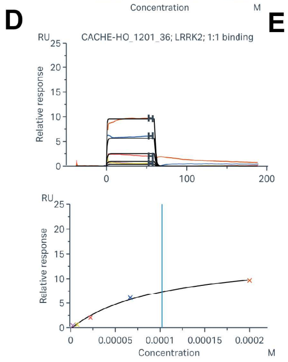

E

Compound Name	Normalized Intensity (kCnt/s)			Laser Power (%)		
	200µM	100µM	50µM	200µM	100µM	50µM
**2% DMSO control**	487	827	1595	100	100	100
CACHE-HO_1201_1	424	810	767	100	100	100
CACHE-HO_1201_18	2340	3364	987	78	100	100
CACHE-HO_1201_23	639	1373	773	100	100	100
CACHE-HO_1201_35	1120	663	1545	100	100	100
CACHE-HO_1201_36	1209	1432	1266	90	95	100

Fig. B4 Experimental results as shared by the CACHE organizers. For compounds **A-D** the SPR data shows an observable dose-dependent binding. However, the quality of the titration curve (left side for each panel, obtained through fitting of SPR sensorgrams right side for each panel) often only allows an approximation of K_D values for the compounds. For each compound **A-E** their respective K_D value, percentage of binding and orthogonal assay results as well as solubility results are reported. For compound **E** only the numerical results were sent due to its low affinity.

Appendix C Supplement List: Exclusion Criteria for Selected Compounds/Undesired Moieties and Properties in Compounds

This derives from several layers of information: on one hand from commonly observed or rarely observed motifs in FDA-approved drugs, on the other hand from scientific publications [3, 4, 38], as well as objective and subjective preferences from medicinal chemists:

- long alkyl chains
- long alkyl chains with further functional groups (“flappy chains”)
- terminal alcohol groups
- polyphenols
- chelating agents
- dicarboxylic acids
- ketones
- β-keto thioether
- α,β-unsaturated amides or ketones
- amino nitriles
- no aromatic groups
- more than two spiro combinations
- flappy lipophilic residues
- phenols
- zwitterions
- pyrroles
- more than two stereocenters
- bi-aryl structures
- silicon-containing compounds
- azides
- more than 50% hetero atoms
- epoxides
- strained rings
- peroxide functional groups
- nitroso groups
- quaternary ammonium groups
- thiol groups
- boron-containing motifs
- hyperconjugated systems
- cyclic imides
- bridged bicyclic structures
- sulfonyl fluorides
- carbodiimides
- nitrilium ions
